# A JUN N-terminal kinase inhibitor induces ectodomain shedding of the cancer-associated membrane protease Prss14/epithin via protein kinase CβII

**DOI:** 10.1074/jbc.RA119.011206

**Published:** 2020-04-02

**Authors:** Joobyoung Yoon, Youngkyung Cho, Ki Yeon Kim, Min Ji Yoon, Hyo Seon Lee, Sangjun Davie Jeon, Yongcheol Cho, Chungho Kim, Moon Gyo Kim

**Affiliations:** ‡ School of Biological Sciences, Seoul National University, Seoul 08826, Korea; § Department of Life Sciences, Korea University, Seoul 02841, Korea; ¶ Department of Biological Sciences, Inha University, Incheon 22212, Korea

**Keywords:** serine protease, shedding, PKC, c-Jun N-terminal kinase (JNK), cell invasion, breast cancer, bioinformatics, cell biology, cell invasion, ectodomain shedding, JNK, protein kinase C β, Prss14/epithin

## Abstract

Serine protease 14 (Prss14)/epithin is a transmembrane serine protease that plays essential roles in tumor progression and metastasis and therefore is a promising target for managing cancer. Prss14/epithin shedding may underlie its activity in cancer and worsen outcomes; accordingly, a detailed understanding of the molecular mechanisms in Prss14/epithin shedding may inform the design of future cancer therapies. On the basis of our previous observation that an activator of PKC, phorbol 12-myristate 13-acetate (PMA), induces Prss14/epithin shedding, here we further investigated the intracellular signaling pathway involved in this process. While using mitogen-activated protein kinase inhibitors to investigate possible effectors of downstream PKC signaling, we unexpectedly found that an inhibitor of c-Jun N-terminal kinase (JNK), SP600125, induces Prss14/epithin shedding even in the absence of PMA. SP600125-induced shedding, like that stimulated by PMA, was mediated by tumor necrosis factor-α–converting enzyme. In contrast, a JNK activator, anisomycin, partially abolished the effects of SP600125 on Prss14/epithin shedding. Moreover, the results from loss-of-function experiments with specific inhibitors, short hairpin RNA–mediated knockdown, and overexpression of dominant-negative PKCβII variants indicated that PKCβII is a major player in JNK inhibition– and PMA-mediated Prss14/epithin shedding. SP600125 increased phosphorylation of PKCβII and tumor necrosis factor-α–converting enzyme and induced their translocation into the plasma membrane. Finally, *in vitro* cell invasion experiments and bioinformatics analysis of data in The Cancer Genome Atlas breast cancer database revealed that JNK and PKCβII are important for Prss14/epithin-mediated cancer progression. These results provide important information regarding strategies against tumor metastasis.

## Introduction

Prss14/epithin (also known as matriptase, suppression of tumorigenicity 14, membrane-type protease-1), a typical member of the type II transmembrane serine protease family, plays important roles in cancer progression and metastasis (1, 2). Overexpression of Prss14/epithin is found in various epithelial cancer types (2). Particularly, Estrogen Receptor (ER)^4^ breast cancer patients with a poor prognosis express higher levels of Prss14/epithin (3). In our earlier report involving pathological examinations, we showed that the prognosis of post-surgery esophageal cancer patients with higher Prss14/epithin expression is very poor (4). Transgenic expression of Prss14/epithin in the mouse causes spontaneous squamous cell carcinomas (5). In contrast, reduced Prss14/epithin expression in a transgenic mouse model clearly impairs tumor progression and metastasis (6). Moreover, down-regulated Prss14/epithin inhibits ErbB2-induced prostate cancer cell invasion (7), and specific inhibitors of Prss14/epithin protease activity impair tumor growth and metastasis (8). We have reported previously that knockdown of Prss14/epithin reduces cell migration (9, 10) and the metastatic ability of breast cancer cells such as 4T1 (9) and EO771 (11). When a specific mAb raised against the activation loop portion of Prss14/epithin is injected into a PyMT breast cancer mouse model, metastasis is reduced significantly (11).

Prss14/epithin has a multidomain structure with a short cytoplasmic N terminus, a single transmembrane region, a sperm protein, enterokinase, and agrin domain (SEA), two complement subcomponent C1r/C1s domains (CUB), four low-density lipoprotein (LDLRA) receptor class A repeats, and a C-terminal serine protease domain (12, 13) and undergoes multiple processing steps before it is fully mature. Its full-length form with a molecular mass of 110-kDa converts to a 92-kDa form after being processed at Gly-149, and this conversion is essential for ectodomain shedding of this protease (14). Previously, we showed that phorbol 12-myristate 13-acetate (PMA) and TGFβ can induce ectodomain shedding of Prss14/epithin (15, 16) by inducing actin cytoskeletal remodeling, which results in filamin-dependent translocation of the protease to the cell–cell contact (17). Filamin appears to be essential for the translocation and shedding of Prss14/epithin (17). Prss14/epithin shedding events induced by PMA or TGFβ occur mainly through the action of tumor necrosis factor-α–converting enzyme (TACE) (15, 16). Although it is still debated whether shedding occurs prior to activation, in the case of TGFβ-induced shedding, shedding precedes activation of the protease (10, 16) suggesting that shedding is possibly required for activation of the protease. Because PMA, a diacylglycerol mimic, acts as activator of the PKC family, and some PKC isoforms have already been recognized for being involved in ectodomain shedding of other transmembrane proteins (18, 19), there is no doubt regarding the involvement of PKCs in ectodomain shedding of Prss14/epithin. However, it is still a mystery which PKC isoforms and other intracellular signaling pathways are involved in shedding of Prss14/epithin. Considering that ectodomain shedding of the protease can profoundly affect the extracellular environment in favor of cancer metastasis (20–22), identification of such signaling pathways will be useful for establishing therapeutic approaches. Moreover, because of the broad biological effects of PKC family members, pinpointing the PKC isoforms responsible for shedding may also be critical for specific targeting of Prss14/epithin ectodomain shedding.

In this study, we show that PKCβII is a key molecule required for shedding of Prss14/epithin induced by PMA and a JNK inhibitor. PMA treatment and JNK inhibition can increase phosphorylation and translocation of PKCβII to the plasma membrane, which could be the essential step for ectodomain shedding of Prss14/epithin. Indeed, inhibition of PKCβII and JNK reduces *in vitro* cell invasion. Finally, bioinformatics analysis revealed that the levels of signaling molecules are correlated with better or worse patient survival. Thus, our finding can provide important information about new therapeutic approaches for cancer patients with high expression of Prss14/epithin.

## Results

### JNK inhibition increases Prss14/epithin shedding

**Figure 1.**
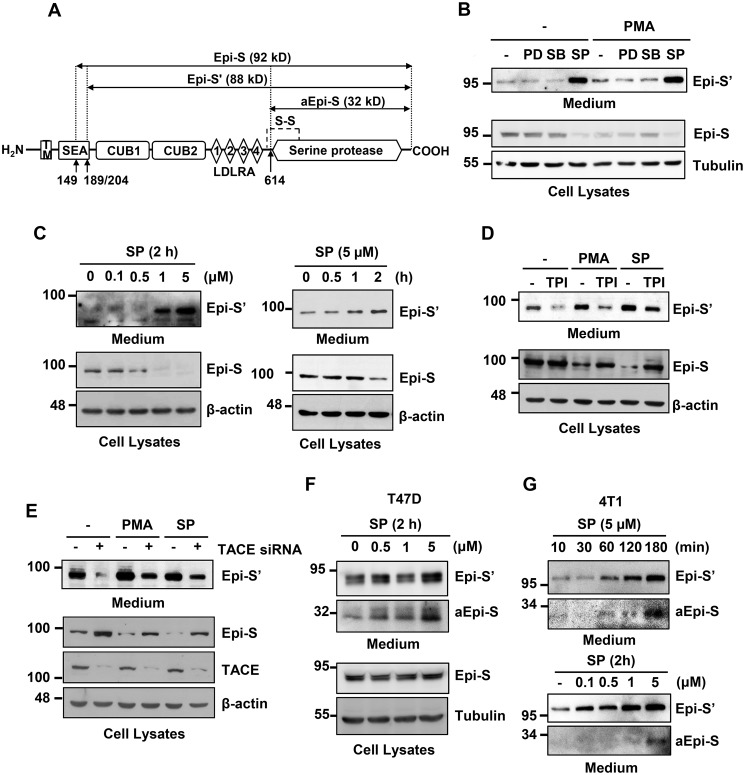
**SP600125 or PMA induce Prss14/epithin ectodomain shedding.** *A*, diagram of Prss14/epithin domain structure and processed forms. Epi-S', Epi-S, aEpi-S are indicated. *B*, effects of MAP kinase inhibitors in Prss14/epithin shedding. 427.1.86 cells were treated with 10 μm PD98059 (*PD*), 20 μm SB203580 (*SB*), and 5 μm SP600125 (*SP*) for 30 min and then with or without 0.5 μm PMA for an additional 2 h. *C*, dose- and time-dependent profiles of Epi-S' and Epi-S. 427.1.86 cells were treated with the indicated concentration of SP600125 for 2 h (*left panel*) and 5 μm SP600125 up to 2 h (*right panel*). *D*, 427.1.86 cells were pretreated with 10 μm TAPI-0 (*TPI*) for 30 min, and then cells were treated with 5 μm SP600125 or 0.5 μm PMA for an additional 2 h. The TACE inhibitor abolished the appearance of Epi-S' while retaining Epi-S in the cell, regardless of shedding induction methods, PMA, and SP600125. *E*, removal of TACE with siRNA abolished shedding of Prss14/epithin. 427.1.86 cells were transfected with 200 nm TACE siRNA for 48 h, starved of serum for 4 h, and then treated with 5 μΜ SP600125 or 0.5 μΜ PMA for 2 h. The control samples were treated exactly the same way except for transfection with nontargeting control siRNA. *F*, SP600125 dose-dependently induced Epi-S' and aEpi-S in T47D cells. SP600125 was treated for 2 h. *G*, SP600125 time-dependently (with 5 μm) and dose-dependently (for 2 h) induced Epi-S' and aEpi-S in 4T1 cells. In all panels, Epi-S' collected from culture medium and other proteins, including Epi-S, from cell lysates were detected by Western blot analysis. Tubulin or β-actin was used for normalization. *TM*, transmembrane domain; *SEA*, sperm protein, enterokinase, and agrin domain; *CUB1*, *CUB2*, complement subcomponent C1r/C1s domain; 1, 2, 3, 4 *LDLRA*, low-density lipoprotein receptor class A repeats.

To investigate signaling pathways involved in PMA-induced Prss14/epithin ectodomain shedding, we first sought to test three main MAPK pathways (extracellular signal-regulated kinase, p38, and JNK) (23) by employing commonly used specific inhibitors in the absence or presence of PMA in 427.1.86 cells. As seen in Figs. 1, *A* and *B* (see Fig. S1 for the full-size blot), in the absence of any pathway-specific inhibitors, PMA slightly increased the shed form of Prss14/epithin (Epi-S') in the conditioned medium but decreased the amount of protein (Epi-S) remaining in the cell lysate. When three inhibitors were used (PD98059 for extracellular signal-regulated kinase, SB203580 for p38, and SP600125 for JNK), SP600125 significantly increased the levels of Epi-S' regardless of the presence of PMA (Fig. 1*B*). The effects of SP600125 on Epi-S' appearance in the medium (and disappearance of Epi-S in the cell lysate) were in a dose- and time-dependent fashion (Fig. 1*C*).

Because we already know that PMA-induced Prss14/epithin shedding is mediated by TACE (15), we tested whether SP600125-induced shedding is also mediated by TACE (Fig. 1, *D* and *E*). Use of a TACE-specific inhibitor and knocking down of TACE expression significantly reduced the amount of Epi-S' in conditioned medium while retaining the amount of cellular Epi-S. In the experiments with 427.1.86 cell line, we rarely observed the smaller activated form in the same blot (data not shown).

We also tested SP600125-induced shedding in other cell types: T47D human breast cancer and 4T1 mouse breast cancer cells. T47D cells showed that SP600125 induced Epi-S' and aEpi-S (30-kDa activated form) in a dose-dependent manner (Fig. 1*F*). The time- and dose-dependent increases in Epi-S' and aEpi-S in 4T1 cells are shown in Fig. 1*G*. The T47D and 4T1 breast cancer cell lines clearly revealed the active form, aEpi-S (with enhanced exposure of the same blot) following the appearance of Epi-S, suggesting that shedding is a general phenomenon and may precede the activation event.

**Figure 2.**
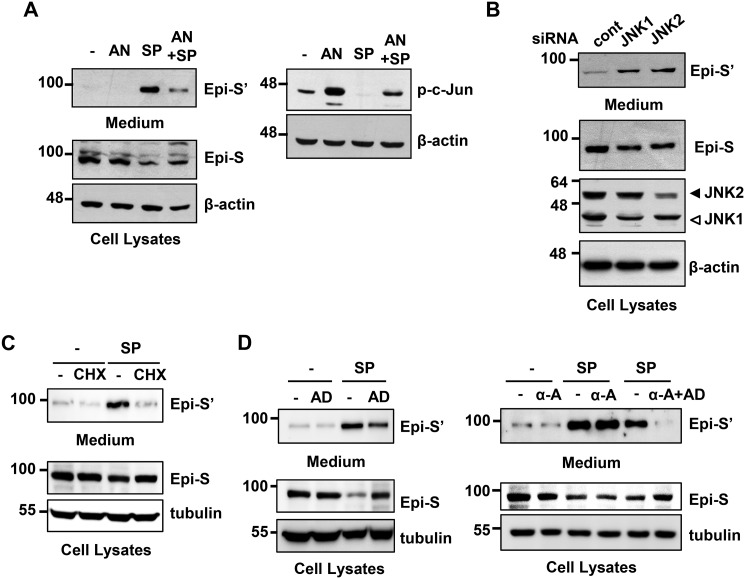
**SP600125-induced Prss14/epithin shedding involves JNK activity and *de novo* synthesis of labile protein.** *A*, 427.1.86 cells were pretreated with 10 μm anisomycin (*AN*) for 30 min and then treated with 5 μm SP600125 (*SP*) for an additional 2 h. Epi-S' collected from culture medium and Epi-S in the cells were detected by Western blot analysis. *B*, suppression of JNKs by transfection with JNK1- or JNK2-specific siRNA induced Prss14/epithin shedding. 427.1.86 cells were transfected with 100 nm of JNK siRNA or nontargeting control (*cont*) siRNA. After 48 h, the medium was replaced and incubated for an additional 2 h before harvesting medium and cells. Control siRNA designed not to target any genes was used as a negative control in knockdown experiments. The band intensities were scanned, and we estimated the degree of percent reduction or -fold increase relative to control samples as described in the text. *C*, effects of cycloheximide (*CHX*) in SP600125-induced shedding. 427.1.86 cells were pretreated with 10 μm cycloheximide and then treated with 5 mm SP600125 for an additional 2 h before harvesting the samples. *D*, effects of pretreatment of actinomycin D (*AD*, 5 μm for 30 min) and/or α-amanitin (α*-A*, 5 μm for 12 h) on SP600125-induced shedding was analyzed as in *A*.

To investigate whether SP600125-induced Prss14/epithin shedding is indeed due to its inhibitory effect on JNK activity, the downstream effects of SP600125 were tested (Fig. 2). SP600125 abolished phosphorylation of p-c-Jun, a target of JNK. It is generally agreed that anisomycin is a JNK activator involved in protein stability and/or transcription (24). To confirm that the effect of SP600125 is specifically mediated by JNK inhibition and not by possible off-target effects of the inhibitor, we attempted to suppress the inhibitory effect by pretreatment with anisomycin and test the effects of SP600125. When anisomycin was used together with SP600125, shedding of Prss14/epithin and phosphorylation of c-Jun were partially affected, suggesting that anisomycin can interfere with SP600125-induced shedding (Fig. 2*A*). In addition, when expression of JNK1 and JNK2 was reduced by specific siRNAs (reductions relative to control samples with nontargeting siRNA were 63.9% for JNK1 and 48.4% for JNK2), ectodomain shedding of Prss14/epithin to the medium was increased 3.0-fold for JNK1 knockdown and 4.8-fold for JNK2 knockdown) (Fig. 2*B*). These results indicate that JNK activity inhibits Prss14/epithin shedding.

To investigate the mechanisms of SP600125-induced Prss14/epithin shedding in more detail, we applied cycloheximide, which is generally considered an inhibitor of protein synthesis. As seen in Fig. 2*C*, cycloheximide abolished SP600125-induced Prss14/epithin shedding. This strongly suggests that Prss14/epithin shedding requires *de novo* synthesis of labile protein(s). When new transcription was interfered with by actinomycin D or α-amanitin pretreatment, SP600125-induced Prss14/epithin shedding was slightly reduced but severely affected by treatment with both reagents together, suggesting that at least some new transcription is required (Fig. 2*D*).

Previously, we showed that actin rearrangement induced by PMA is essential for translocation and shedding of Prss14/epithin (17). SP600125, similar to PMA, induced actin to rearrange to form cortical actin filaments. This rearrangement was abolished by additional anisomycin treatment (Fig. S2).

### PKCβII is involved in PMA- and SP600125-induced Prss14/epithin shedding

**Figure 3.**
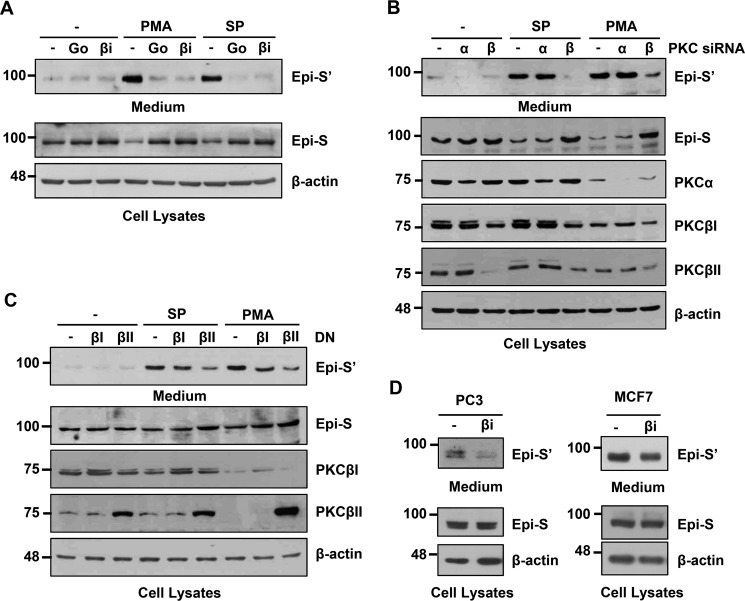
**PKCβII is responsible for PMA- and SP600125-induced shedding of Prss14/epithin.** *A*, 427.1.86 cells were pretreated with 5 μm Go6976 (*Go*) or 1 μm PKCβ-selective inhibitor (β*i*) before treatment with 5 μm SP600125 (*SP*) or 0.5 μm PMA for 2 h. *B*, PKCα or PKCβ knockdown effects on SP600125- or PMA-induced Prss14/epithin shedding. 427.1.86 cells were transfected with 200 nm PKCα or PKCβ siRNA or nontargeting control siRNA for 48 h and then treated with 5 μm SP600125 or 0.5 μm of PMA for an additional 2 h. PKCβ knockdown abolished SP600125- and PMA-induced shedding of Prss14/epithin. *C*, 427.1.86 cells were transfected with 1 μg/ml of the DN form of PKCβI and PKC PKCβII for 72 h. Control cells were transfected with an empty vector. DN forms of PKCβII inhibited shedding. *D*, PKCβ inhibition of PRSS14 shedding in two human cell lines. PC3 prostate cancer cells and MCF7 breast cancer cells were maintained in serum-free medium overnight and then treated with 1 μm PKCβ inhibitor for an additional 6 h before testing.

We tried to identify the PKC isoforms involved in PMA- and/or SP600125-induced Prss14/epithin shedding by using PKC inhibitors, siRNAs, and dominant-negative (DN) forms (Fig. 3). Treatment with a broad-spectrum PKC inhibitor, Go6976, and a PKCβ selective inhibitor (βi) significantly inhibited PMA- and SP600125-induced shedding of Prss14/epithin in 427.1.86 cells (Fig. 3*A*). Both inhibitors decreased Epi-S' in the medium, whereas Epi-S levels in the cell lysate were similar to those of Epi-S'. Knockdown studies using PKCα and β siRNAs were then carried out (Fig. 3*B*). Only PKCβ, but not PKCα siRNA, affected SP600125- and PMA-induced shedding. Because PKCβI and PKCβII use the same message, the levels of PKCβI and PKCβII proteins were decreased when cells were treated with PKCβ siRNA. PMA treatment also reduced PKC levels, which is known as PMA-induced down-regulation following activation (25). However, treatment with 5 μm SP600125 for 2 h did not severely affect the expression levels of these PKC isoforms. From these results, it was concluded that SP600125- and PMA-induced Prss14/epithin shedding was primarily mediated by PKCβ, not by PKCα (Fig. 3*B*). Next, DN forms of PKCβ isoforms were tested. Overexpression of dnPKCβII cDNA inhibited PMA- or SP600125-induced Prss14/epithin shedding whereas overexpression of dnPKCβI cDNA did not (Fig. 3*C*). The PKCβ inhibitor also reduced SP600125-induced Prss14/epithin shedding in PC3 and MCF7 cells (Fig. 3*D*). These results indicate that PKCβII is critical for PMA- and SP600125-induced Prss14/epithin shedding.

### SP600125 induces activation of PKCβII and membrane translocation

**Figure 4.**
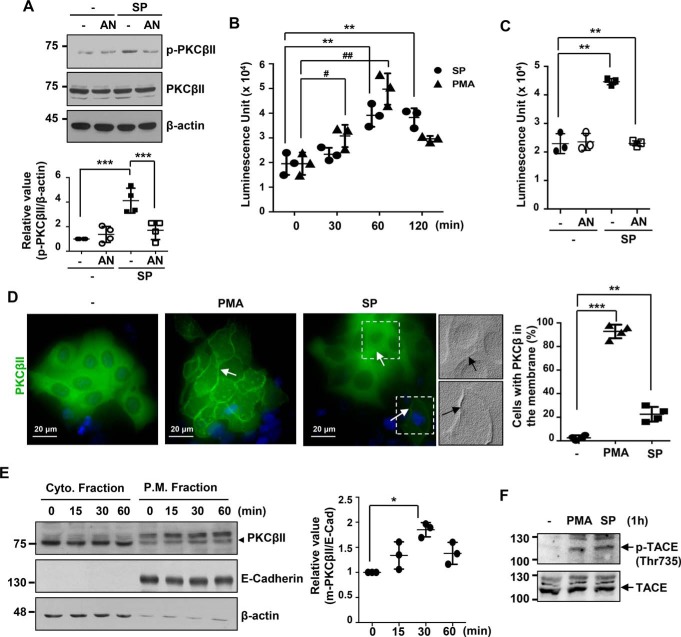
**JNK inhibition increases PKCβII activity, translocation into the membrane, and TACE phosphorylation.** *A*, phosphorylation of PKCβII by the JNK inhibitor. 427.1.86 cells were pretreated with 10 μm anisomycin (*AN*) for 30 min and then treated with 5 μm SP600125 (*SP*) for an additional 1 h. SP600125 treatment induced PKCβII phosphorylation. Relative values of band intensity are expressed as means ± S.D. of four independent experiments. ***, *p* < 0.001. *B*, kinetics of SP600125- or PMA-induced PKCβII activity. 427.1.86 cells were incubated with 5 μm SP600125 or 0.5 μm PMA for 0, 30, 60, and 120 min. After immunoprecipitation with PKCβII antibody, PKCβII activities were determined using the ADP-Glo^TM^ kinase assay kit. All values are expressed as means ± S.D. **, *p* < 0.01; #, *p* < 0.05; ##, *p* < 0.01; *n* = 3. *C*, Enzymatic activity of PKCβII was induced by SP600125 alone not by anisomycin combination to SP600125. 427.1.86 cells were pretreated with 1 μm anisomycin for 30 min and then threated with 5 μm SP600125 for an additional 1 h. All values are expressed as means ± S.D. **, *p* < 0.01; *n*=3. *D*, immunofluorescent staining of PKCβII-overexpressing 427.1.86 cells. Cells were transfected with 1 μg/ml of PKCβII WT cDNA for 48 h and then treated with 5 μm SP600125 or 0.5 μm PMA for 1 h. Immunofluorescence staining was performed with anti PKCβII polyclonal antibody (1:50) followed by FITC-conjugated anti rabbit IgG antibody (1:200). For nucleus staining, cells were incubated with DAPI for 10 min. Membrane localization of PKCβII is indicated by *arrows*. Images of two cells treated with SP600125 were stylized by embossing the appearance of the signal intensities using Adobe Photoshop. The graph indicates the percentage of cells with PKCβII localized in the membrane from four independent experiments. All values are expressed as means ± S.D. **, *p* < 0.01; ***, *p* < 0.001. *E*, membrane localization of PKCβII by cellular fractionation. 427.1.86 cells were treated with 5 μΜ SP600125 up to 60 min, and then PKCβII in cytosolic and membrane fractions was examined. Relative values of band intensity are expressed as means ± S.D. for three independent experiments. The *arrowhead* indicates PKCβII. *, *p* < 0.05. *F*, phosphorylation of TACE by shedding inducers. In the cells treated with 0.5 μm PMA or 5 μm SP600125 for 1 h, phosphorylation of TACE was analyzed by Western blot analysis using an antibody specific for phosphorylated TACE (Thr-735).

Signaling further downstream of PKCβII, such as activation and translocation of PKCβII, was then investigated with SP600125 in 427.1.86 cells (Fig. 4). SP600125 increased phosphorylation of PKCβII at serine 660 (a hallmark of the active form) whereas additional anisomycin pretreatment did not (Fig. 4*A*). In all samples, the total amounts of PKCβII were not changed upon treatment. In addition, the activity of PKCβII in immunoprecipitated samples treated with SP600125 peaked after 1 h (Fig. 4*B*) and was reduced by anisomycin (Fig. 4*C*).

The location of PKCβII after SP600125 treatment was in the plasma membrane (Fig. 4, *D* and *E*). Because levels of endogenous PKCβII protein were insufficient to investigate cellular localization on a small scale, cellular location was investigated using overexpression of PKCβII. Thus, cells were transfected with PKCβII cDNA, and then cellular localization of PKCβII was determined by specific antibody staining using immunofluorescence microscopy and stylized image transformation. Indeed, SP600125 induced translocation of PKCβII to the membrane (Fig. 4*D*). PKCβII in SP600125-treated cells clearly located in the cell-to-cell contact among clustered cells or in the plasma membrane of an isolated cell. When the fractions of cells with PKCβII appearing in the membrane were calculated, almost all cells, if not all cells, in PMA-treated samples showed complete membrane localization, but only 20% of cells in SP600125-treated samples showed clear membrane localization. Using subcellular fractionation, as measured by the ratio of phosphorylated PKCβII over E-cadherin of each fraction, the peak of PKCβII in the membrane fraction was obtained at 30 min (Fig. 4*E*).

In Fig. 4*F*, we show the appearance of phosphorylated TACE upon PMA or SP600125 treatment. Phosphorylation at threonine 735 of TACE, which reflects activation of the enzyme, was increased by PMA and SP600125. From these results, we concluded that TACE phosphorylation is common downstream of PMA and SP600125.

### PKCβII plays critical role in PMA- or SP600125-induced cell invasion

**Figure 5.**
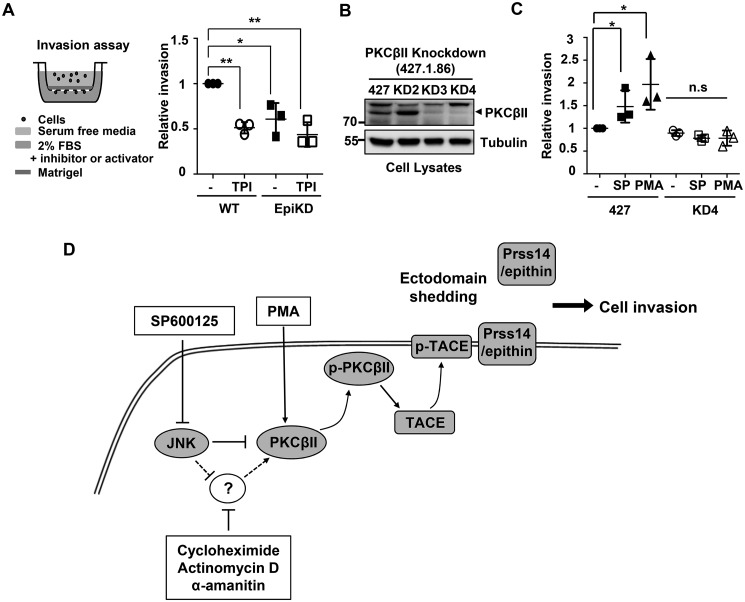
**PKCβII is critical for PMA- or SP600125-induced cell invasion.** *A*, schematic of the serum induced invasion assay (*left panel*). Invasion of 4271.86 cells depends on TACE activity and Prss14/epithin expression. After serum starvation for 12 h, cells were plated in the upper chamber, and DMSO or 20 μm TAPI-0 (*TPI*) was added to the lower chamber. Invaded cells on the underside of the membrane after 24 h were stained with crystal violet and counted. The *left panel* shows the -fold change of the average number of invaded cells in five randomly selected fields. All values are expressed as means ± S.D. *, *p* < 0.05; **, *p* < 0.01; *n*=3. *B*, PKCβII was knocked down in 427.1.86 cells using PKCβII siRNA (*KD2*, *KD3*, and *KD4*). Expression of PKCβII was reduced in KD3 and KD4. *C*, invasive activity in PMA- and SP600125-treated 427.1.86 and PKCβII knockdown cells. The graph shows the -fold change of the average number of invaded cells on the underside of the membrane after 24 h in the presence or absence of PMA or SP600125. Cells in five different microscopic fields were counted. All statistical analyses were performed using unpaired two-tailed Student's *t* test. *, *p* < 0.05; *ns*, not significant. *Error bars*, mean ± S.D. (*n* = 3). *D*, model of intracellular signaling events and modulation by SP600125 and PMA during ectodomain shedding of Prss14/epithin.

Previously, we and others showed that Prss14/epithin is important for cell invasion (10, 26, 27). Serum is also an important factor for inducing Prss14/epithin shedding. Consistently, the invasiveness of 427.1.86 cells was decreased in both Prss14/epithin knockdown cells and TACE inhibitor-treated cells (Fig. 5*A*). To study the role of PKCβII in cell invasion, we generated PKCβII-specific knockdown cell lines (Fig. 5*B*). As seen Fig. 5*C*, the degrees of 427.1.86 cell invasion induced by the serum gradient was increased upon SP5200125 or PMA treatment. However, the invasive nature of PKCβII knockdown cells under the same condition was abolished, even in the presence of SP5200125 or PMA (Fig. 5*C*).

From these results, we established a model of how SP600125 induces ectodomain shedding of Prss14/epithin. SP600125 suppresses the inhibitory effect of JNK on PKCβII, and the SP600125-mediated increase in PKCβII activity may require a labile, not yet identified regulator of PKCβII (Fig. 5*D*). PKCβII can be activated after PMA or JNK inhibition, either directly or indirectly, affecting TACE activation. PKCβII, TACE, and Prss14/epithin are translocated to the membrane; then cleavage of Prss14/epithin occurs at the membrane, increasing cell invasion.

### PKCβII and JNKs as prognostic markers in metastatic breast cancer

**Figure 6.**
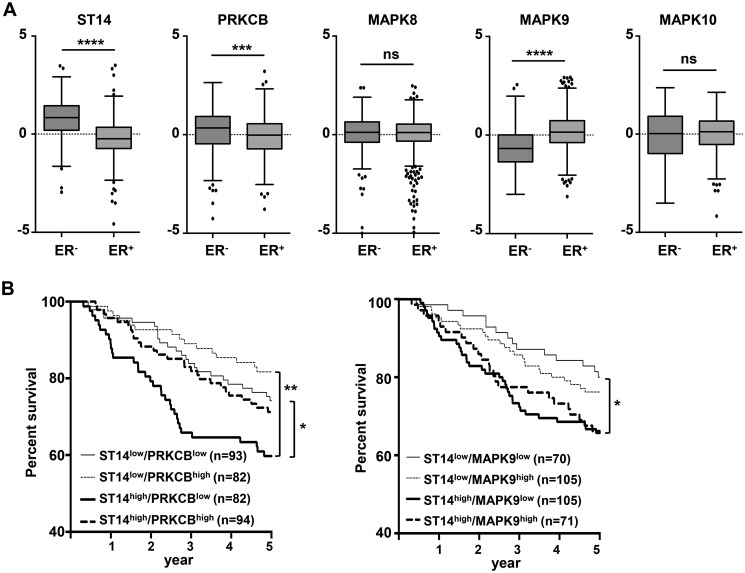
**PKCβII and JNKs are good prognostic markers in metastatic breast cancer together with ST14.** *A*, box plots presenting mRNA expression of the indicated genes in ER^−^ and ER^+^ breast cancer patients from TCGA datasets. Comparisons were analyzed by unpaired two-tailed Student's *t* test. *B*, survival analysis of four breast cancer patient groups divided by expression levels of ST14 and PRKCB (*left panel*) or MAPK9 (*right panel*). *p* values were calculated using log rank statistics. *, *p* < 0.05; **, *p* < 0.01; ***, *p* < 0.001; ****, *p* < 0.0001; *ns*, not significant.

To evaluate the clinical significance of PKCβII and JNKs in cancer patients, we utilized the publicly available TCGA breast cancer RNA-Seq database (Fig. 6). The expression levels of Prss14/epithin (ST14), PKCβ (PRKCB), and three JNKs (MAPK8, MAPK9, and MAPK10) in more aggressive ER-negative (ER^−^) and less aggressive ER-positive (ER^+^) breast cancer patients were compared individually in the plot (Fig. 6*A*). ST14 and PRKCB levels were higher in ER^−^ patients, as expected. Among the MAPKs, MAPK9 (JNK2) exhibited significant differences between two groups. MAPK9 levels were higher in ER^+^ patients.

In a survival curve analysis (Fig. 6*B*), using a combination of ST14 and PRKCB, ST14^high^ and PRKCB^high^ group patients showed significantly lower survival compared with ST14^low^ PRKCB^low^ and ST14^low^ PKRCB^high^ group patients, suggesting that ST14 is a stronger parameter when used with PRKCB. MAPK9 did not affect patient survival in a statistically significant way, although ST14^low^ MAPK9^low^ appeared to have the best survival.

## Discussion

In this study, we showed upstream signaling of induced Prss14/epithin shedding and the significance and application of the resulting biology. Prss14/epithin shedding was induced by JNK inhibition by SP600125 as well as PMA, which shares downstream molecules such as PKCβII and TACE through phosphorylation and translocation (see the summary model in Fig. 5*C*).

### JNK inhibition in addition to PMA can mediate Prss14/epithin shedding through PKCβII and TACE

SP600125- or PMA-induced shedding is mediated by TACE. We already showed that PMA- and TGFβ-induced Prss14/epithin shedding depends on TACE (15, 16). Although Prss14/epithin shedding can be blocked by serine protease inhibitors such as ecotin (17, 28), we still believe that shedding is mediated by TACE, as tested by specific inhibition and knockdown experiments (Fig. 1, *D* and *E*). It is likely that shedding precedes activation, as shown in Fig. 1, *F* and *G*. The activated form of 30-kDa appeared more slowly in the medium than the shed form. In addition, we hardly observed the 30-kDa form in cell lysates (data not shown). In the case of TGFβ-induced Prss14/epithin shedding, we showed that the kinetics of Prss14/epithin activity were slower than those of Prss14/epithin shedding (16).

JNK activity, as revealed by p-c-Jun, appeared to indirectly affect Prss14/epithin shedding (Fig. 2). JNK inhibition by SP6200125 was partially affected by anisomycin, a known JNK activator and a general inhibitor of protein synthesis. Knocking down JNK1 or JNK2 expression using siRNAs also induced Prss14/epithin shedding (Fig. 2*B*). SP6200125-induced Prss14/epithin shedding was also affected by cycloheximide in addition to the transcription inhibitors α-amanitin and actinomycin D. These observations suggest that JNK plays a role in Prss14/epithin shedding through labile protein degradation or diminished protein stability.

Among conventional PKC isotypes that are known for being activated by PMA, PKCβII was found to be involved in PMA- and SP600125-induced shedding of Prss14/epithin (Fig. 3). Experiments with isotype-specific inhibitors, specific knockdown, and DN forms revealed that PKCβII is the key player in Prss14/epithin shedding induced by JNK inhibition or PMA exposure. JNK inhibition alone is sufficient to induce translocation of PKCβII to the membrane. Inhibition of JNKs increases phosphorylation of PKCβII at serine 660 (Fig. 4*A*). Serine 660 of PKCβII resides in a C-terminal hydrophobic region and controls PKCβII folding and stability (29), activating PKCβII. SP600125, in fact, increased the activity of PKCβII (Fig. 4, *B* and *C*) and induced TACE activation similar to PMA (Fig. 4*D*). These results are incorporated in the model presented in Fig. 5*C*.

In our earlier studies, we showed that actin rearrangement from stress fibers to the cortical actin form is essential for Prss14/epithin shedding (17). This actin rearrangement involves filamin, which can function as a vessel to bring proteins together (30). Prss14/epithin and TACE are brought to membranes by filamin upon PMA activation and interact together in complexes (17). Thus, TACE can cleave Prss14/epithin at the membrane. Because SP600125 induces cortical actin formation, and PKCβII translocates to the membrane, we suspect that PKCβII may also interact with filamin. However, to date, there are no data to support this idea, although PKCα and PKCϵ isoforms have been shown to bind to filamin (31, 32). There is evidence that actin cytoskeleton remodeling is associated with JNK activity. Actin stress fibers and the associated shear stress mediate inhibition of JNKs in vascular endothelium (33). TGFβ-induced actin stress fibers promote activity of JNK in human mesangial cells (34). Inhibition of JNK induces rearrangement of actin stress fibers to cortical actin filaments in 427.1.86 cells by interaction (Fig. S2). Therefore, it is conceivable that inhibition of JNK may lead to interactions to form complexes of F-actin, filamin, and PKCβII.

### Implication of PKCβ and JNK as prognosis markers and as therapeutic targets for metastatic cancer

The biological significance of Prss14/epithin shedding is intriguing at this point; although activity clearly takes important parts in tumorigenesis and homeostasis of normal epidermal barrier (6, 35–37). From the results obtained using PKCβII knockdown cell lines that had lost the ability to induce invasion (Fig. 5, *A* and *B*), we now understand that PKCβII-mediated shedding is necessary for cell invasion.

In our earlier careful analysis of TCGA breast cancer patients, we showed that Prss14/epithin (ST14) is an excellent prognostic marker for highly metastatic ER^−^ breast cancer (3). In this study, we show that the expression profiles of PRKCB and ST14 are reasonably well correlated (Fig. 6*A*). The levels of PRKCB were higher than those of ST14 in ER^−^ patients, and MAPK9 (JNK2) levels were lower in the same group. In the survival analysis (Fig. 6*B*), PRKCB collaborated with ST14 in terms of the poorest survival of patients. ST14^low^ PRKCB^low^ patients have the best survival. Taking these analyses into consideration, we propose that PRKCB and MAPK9 can be additional prognosis markers and can be used in precision medicine for breast cancer patients.

JNK has previously been considered a target for cancer therapy using kinase inhibitors (38). However, there are complications and complexities when using such applications (39). It is important to note that JNK negatively regulates PKCβ downstream of Prss14/epithin shedding and that JNK2 expression is lower in more aggressive cancers. Therefore, these observations are critical when considering JNK inhibition as a therapeutic approach, at least in breast cancer.

## Experimental procedures

### Cell culture

Cells were maintained in DMEM with 10% FBS, 100 units/ml penicillin, and 100 μg/ml streptomycin. For drug treatments, 90% confluent cells were serum-starved for 4 h and then treated with 0.5 μm PMA (Sigma) or SP600125 (Merck Millipore, Billerica, MA). A PKCβ-selective inhibitor (539654, Merck Millipore), anisomycin (Sigma), and TAPI-0 (Merck Millipore) were used for 30 min before drug treatment. Cycloheximide, α-amanitin, and actinomycin D were purchased from Sigma. For overexpression or knockdown of specific proteins, transfection of cDNA or siRNA was performed. When cells reached 30%–40% confluence, cells were transfected with JNK1 siRNA, JNK2 siRNA, PKCα siRNA, PKCβ siRNA, and TACE siRNA (Santa Cruz Biotechnology, Dallas, TX), WT PKCβII cDNA, DN PKCβI (K371R), and DN PKCβII (K371R) cDNA (Addgene, Cambridge, MA) using Lipofectamine 2000 (Invitrogen) for 48–72 h. Control siRNA (catalog no. sc-37007, Santa Cruz Biotechnology), designed not to target any gene, was used as a negative control in knockdown experiments.

### Western blot analysis

Total cell lysates were extracted with RIPA buffer containing 50 mm Tris-HCl (pH 8.0), 150 mm NaCl, 1% Nonidet P-40, and 0.1% SDS supplemented with protease and phosphatase inhibitors. Soluble Prss14/epithin released into cell culture medium was obtained through protein precipitation using TCA solution (10% final concentration). Protein lysates were separated by SDS-PAGE and transferred to a nitrocellulose or PVDF membrane. The membrane was blocked with 5% nonfat dry milk in TBS, and proteins were detected using specific antibodies: mAb5 (40), anti PKCβII polyclonal antibody (catalog no. sc-13149, Santa Cruz Biotechnology), anti-phospho-specific PKCβII (Ser-660) polyclonal antibody (catalog no. sc-365463, Santa Cruz Biotechnology), anti-JNK1/2 mAb (catalog no. sc-7345, Santa Cruz Biotechnology), anti-E-cadherin polyclonal antibody (catalog no. sc-8426, Santa Cruz Biotechnology), anti-TACE polyclonal antibody (catalog no. ab13535, Abcam), anti-phospho-specific TACE (Thr-735) polyclonal antibody (catalog no. ab182630, Abcam), and anti-β-actin mAb (catalog no. A1978, Sigma). Blots were developed using a peroxidase-conjugated secondary antibody and an ECL system. Relative band intensities were evaluated using ImageJ software (National Institutes of Health).

### Subcellular fractionation

Cells were plated in a 100-mm dish at 2.5 × 10^6^ cells and harvested with Tris buffer containing 10 mm Tris-HCl (pH 8.0), 10 mm NaCl, 1 mm KH_2_PO_4_, 5 mm NaHCO_3_, 1 mm CaCl_2_, and 0.5 mm MgCl_2_ supplemented with protease and phosphatase inhibitors. After sonication on ice, nuclei and debris were eliminated by centrifugation at 7,500 rpm for 5 min. Supernatants containing cytosol and plasma membrane were centrifuged at 34,000 rpm for 60 min (Sorvall Ultracentrifuge OTD-combi). Cytosol supernatants were collected, and crude plasma membrane pellets were resuspended with RIPA buffer and centrifuged at 34,000 rpm for 15 min. Plasma membrane supernatants were collected. To verify cross-contamination, β-actin and E-cadherin were detected by Western blot analysis.

### Immunocytochemistry

Immunocytochemistry was performed as described previously (16). F-actin and PKCβII were detected with rhodamine-conjugated phalloidin (Molecular Probes) and anti-PKCβII polyclonal antibody, respectively. Cells labeled with anti-PKCβII polyclonal antibody were incubated with FITC-conjugated anti-rabbit IgG (Santa Cruz Biotechnology). Cells were observed with a fluorescence microscope (Axio-observer Z1m, Zeiss, Germany). Fluorescence images were captured and processed with Axio-vision imaging software for microscopy.

### Immunoprecipitation, PKCβII activity assay, and TACE phosphorylation

PKCβII activity was determined by modifying an immune complex kinase assay method described elsewhere (41). Briefly, cells were lysed with Tris lysis buffer containing 20 mm Tris-HCl (pH 7.5), 1 mm EDTA, 1 mm EGTA, 150 mm NaCl, 1% Triton X-100, 2.5 mm sodium pyrophosphate, 1 mm β-glycerol phosphate, protease inhibitors, and phosphatase inhibitors. The cell lysates were precipitated with anti-PKCβII polyclonal antibody and protein A–Sepharose beads (GE Healthcare). PKCβII kinase activity was determined by ADP-Glo^TM^ kinase assay kit (Promega). Luciferase values were detected using a Glomax 96 microplate luminometer (Promega). Statistical analyses were performed using Sigma Plot software. Comparisons between two groups were performed by Student's *t* test, and results are expressed as means ± S.D. To investigate phosphorylation of TACE, cell lysates were analyzed by Western blot analysis using anti-phospho-TACE antibody (Thr-735) and anti-total TACE antibody.

### Cell invasion assay

The invasion assay was performed using BioCoat Matrigel invasion chambers (Corning) according to the manufacturer's instructions. 427.1.86, 427-PKCβII-KD4, and 427-EpiKD (9) cells were incubated with serum-free medium for 12 h. Cells (2 × 10^5^) were seeded on the upper side of a BioCoat Matrigel invasion chambers. The lower chamber was filled with DMEM containing 2% FBS with PMA (1 μm), SP600125 (5 μm), or TAPI-0 (20 μm). After 24 h, cells on the lower surface of the membrane were fixed with 100% methanol for 10 min and stained with 0.2% crystal violet for 5 min. Invading cells were counted under an Axioimager M1 in five random fields. The total number of cells was divided by the number of counted fields in each assay.

### Analysis of TCGA datasets

The Cancer Genome Atlas (TCGA) breast cancer patient data were downloaded using the Broad Institute TCGA Genome Data Analysis Center (2016) web portal, which was developed for automated analyses of TCGA data for general users (42). For comparison of gene expression between ER^−^ and ER^+^ groups, box plots were generated using GraphPad Prism 7. Comparisons were analyzed by unpaired two-tailed Student's *t* test. For the 5-year survival rate, Kaplan–Meier survival analysis was performed using TCGA breast cancer data from patients who had not lost contact for five years. *p* values were calculated using a log rank (Mantel–Cox) test, and the hazard ratio was determined by the Mantel–Haenszel method.

### Data availability

All data are contained in this manuscript.

## Author contributions

J. Y., H. S. L., Yongcheol Cho, C. K., and M. G. K. conceptualization; J. Y., Youngkyung Cho, K. Y. K., M. J. Y., H. S. L., S. D. J., Yongcheol Cho, and C. K. investigation; J. Y., H. S. L., Yongcheol Cho, and C. K. methodology; J. Y. writing-original draft; Youngkyung Cho visualization; Youngkyung Cho, C. K., and M. G. K. writing-review and editing; M. G. K. supervision; M. G. K. funding acquisition.

## Supplementary Material

Supporting Information

## Footnotes

4 The abbreviations used are:

ER: Estrogen receptor
PMA: phorbol 12-myristate 13-acetate
TGF: transforming growth factor
TACE: tumor necrosis factor-α–converting enzyme
JNK: c-Jun N-terminal kinase
MAPK: mitogen-activated protein kinase
DN: dominant-negative
cDNA: complementary DNA
TCGA: The Cancer Genome Atlas.
