# Supplementary material for: A JUN N-terminal kinase inhibitor induces ectodomain shedding of the cancer-associated membrane protease Prss14/epithin via protein kinase CβII
Source: J Biol Chem. 2020 Apr 2;295(20):7168–77. doi: 10.1074/jbc.RA119.011206 (PMC7242708; doi:10.1074/jbc.RA119.011206)
Supplement: Supporting Information [file supp_295_20_7168__index.html]

A JUN N-terminal kinase inhibitor induces ectodomain shedding of the cancerassociated membrane protease Prss14/epithin via protein kinase CβII — PKCβII in Prss14/epithin shedding induces cell invasion — A JUN N-terminal kinase inhibitor induces ectodomain shedding of the cancer-associated membrane protease Prss14/epithin via protein kinase CβII — PKCβII in Prss14/epithin shedding induces cell invasion — Supporting Information 

# A JUN N-terminal kinase inhibitor induces ectodomain shedding of the cancer-associated membrane protease Prss14/epithin via protein kinase CβII

## Supporting Information

- Supporting Information (to be published online) - Supporting information included
