# Supplementary material for: A JUN N-terminal kinase inhibitor induces ectodomain shedding of the cancer-associated membrane protease Prss14/epithin via protein kinase CβII
Source: J Biol Chem. 2020 Apr 2;295(20):7168–77. doi: 10.1074/jbc.RA119.011206 (PMC7242708; doi:10.1074/jbc.RA119.011206)
Supplement: Supporting Information [file supp_RA119.011206_156046_2_supp_493428_q71cc7.pdf]

Supporting information

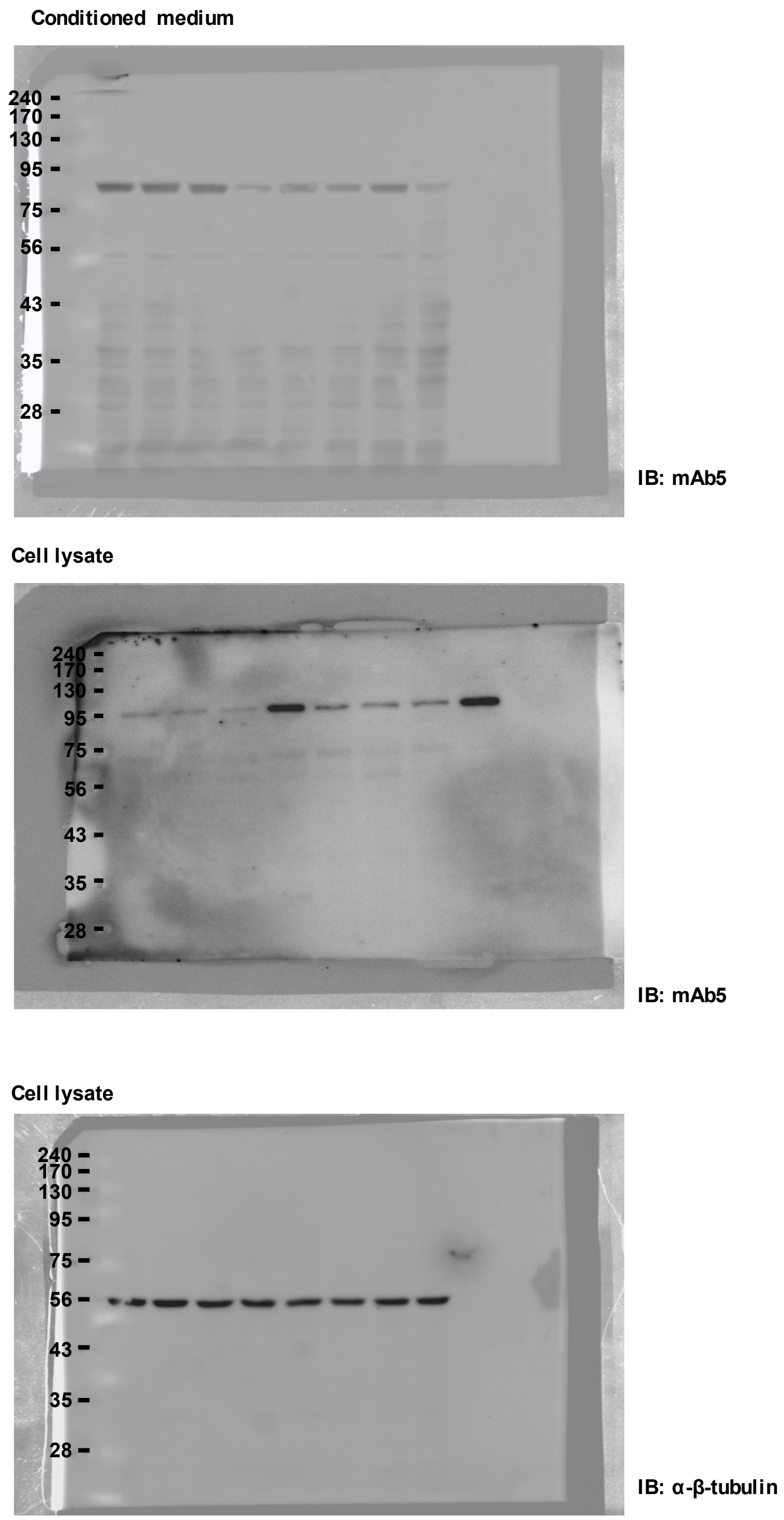

Figure S1. Uncropped full size blot images of Fig. 1B.

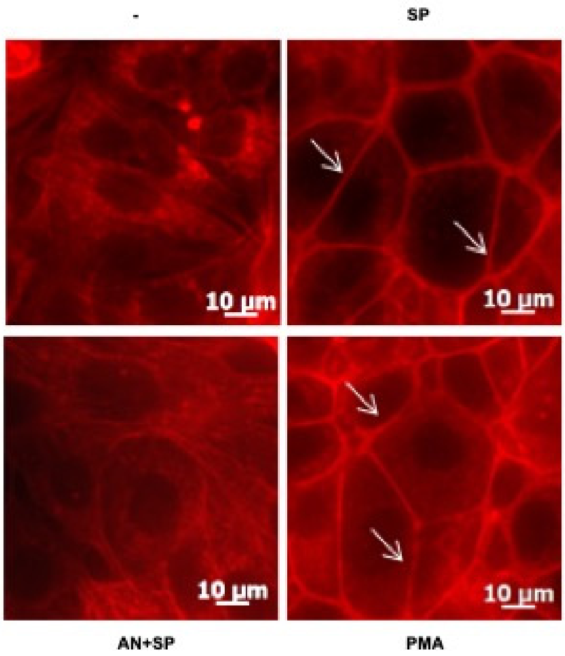

Figure S2. Actin rearrangement induced by SP600125. SP, 5 µM SP600125 treated sample, AN+SP, anisomycin (10 µM) and SP600125 (5 µM) treated sample, PMA, 0.5 µM PMA treated sample. 427.1.86 cell treated with SP600125 shows cortical actin (arrows) formation while of anisomycin cotreatment to SP600125 does not. As a positive control, PMA was used. Cells were fixed with formaldehyde (3.7%) and F-actins were visualized with Rhodamine-conjugated phalloidin.
